# Pairwise comparison based failure mode and effects analysis (FMEA)

**DOI:** 10.1016/j.mex.2020.101007

**Published:** 2020-07-23

**Authors:** Edina Kulcsár, Tamás Csiszér, János Abonyi

**Affiliations:** a University of Pannonia, MTA-PE "Lendület" Complex Systems Monitoring Research Group, POB 158, 8200 Veszprém, Hungary; b Óbuda University, Sándor Rejtő Faculty of Light Industry and Environmental Protection Engineering, Budapest, Doberdó street 6, 1034, Hungary

**Keywords:** Risk assessment, Consistency, Analytic hierarchy process (AHP)

## Abstract

The proposed method supports the determination of severity (S), occurrence (O), and detection (D) indices of Failure Modes and Effects Analysis (FMEA). Previously evaluated and previously not studied risks are compared in pairwise comparison. The analysis of the resulted pairwise comparison matrix provides information about the consistency of the risk evaluations and allows the estimation of the indices of the previously not evaluated risks. The advantages of the method include:

• The pairwise comparison facilities the identification of risks that are otherwise difficult to evaluate
• The inconsistency of existing FMEA studies can be highlighted and systematically reduced
• The method can be generalized about a wide range of grading problems

## Graphical abstract

**Figure:**
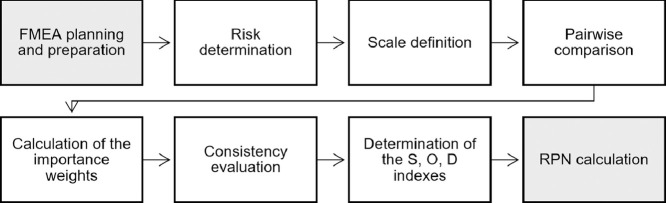

Specifications Table

**Table:** 

Subject Area*Engineering*	*Select one of the following subject areas:Engineering*
More specific subject area:	*Quality engineering*
Method name:	*Pairwise comparison based FMEA (PC-FMEA)*
Name and reference of original method:	*Failure mode and effects analysis (FMEA)*
Resource availability:	*Excel file demonstrating the applicability of the method*

## Method details

### Background

Failure Mode and Effects Analysis (FMEA) is a method of identifying and fully understanding the potential causes and effects of a failure on the system or on end users, for a given product or process [1] and preventing the problems before they occur [2]. FMEA is a widely used method, particularly in the automotive industry [3] of formalizing a complete set of actions that will reduce the risk associated with a system or manufacturing/assembly process. In FMEA, each failure mode is ranked in order of the Risk Priority Number (RPN) which is calculated by multiplying the values of three risk factors of a failure namely the severity (S), probability of occurrence (O) and probability of detection before the effects of the failure are realized (D) [4], and is represented by numbers, generally between 1 (used in the case of no risk) and 10 (used in the worst case).

The team determines the severity and likelihood of the failure occurring or being detected using scales by carefully reviewing the criteria to establish the rankings. This assessment of the rankings should be as objective as possible and take into account the past field history of similar items, previous test results, experience with comparable systems as well as other sources of information. A subjective element of this ranking will always be present as the FMEA is usually conducted on new designs and processes. However, the FMEA team should endeavour to be as objective as possible by using the criteria from the scales to help determine the appropriate ranking. The evaluation of the failure modes is conducted by the FMEA team based on their experience and knowledge. Therefore, separate assessments can be provided by different engineers, which in most cases are more accurate. The scales are not that precise and understandable so compiling a consistent classification is also difficult.

As FMEA is a hierarchical multi-criteria decision-making method, hierarchically structured risks can be prioritized by the Analytic Hierarchy Process (AHP) [5] based pairwise comparison [6]. The concept of AHP and other pairwise comparison based techniques is based on the fact that it is much easier to make comparisons than direct evaluations. AHP [7] and other MCDM (Multiple-criteria decision-making) [8] methods have already been applied to improve FMEA studies by determining the weights of the S, O and D numbers to provide more interpretable RPNs and select a reference table that better reflects the judgments of the FMEA team [9]. Fuzzy approaches [10] including fuzzy AHP was also used to develop the limitations of the FMEA [11], especially for the RPN calculation [12] and for a more accurate risk assessment [13]. These approaches are too difficult to apply, do not result in crisp RPN number, so the resulted FMEAs may have shortcomings [14]. As it has been presented, several examples are available in the literature about the fuzzy pairwise comparison and FMEA. The fuzzy pairwise evaluation is difficult to interpret and implement, the identification and the analysis of the models are not straightforward, which makes these works difficult to be applied in the industry where the aim is to work based on standard FMEA studies and generate crisp RPN numbers. Industrial practitioners require easily interpretable and implementable FMEA methods. As the proposed approach is based on the easily interpretable differences of the O, S, D indices and utilises simple arithmetic operations instead of the calculation of the eigenvector (as it is needed in the classical AHP), it is easily implementable in Excel and the application of the proposed tool does not require any additional knowledge. *The PC-FMEA method*.

**Fig. 1:**
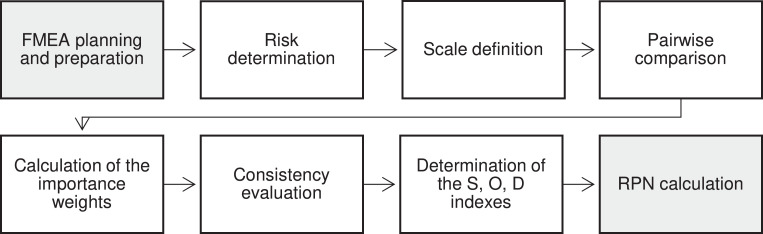
The process of the proposed method incorporated into the standard FMEA process (denoted by grey boxes).

The steps of the methodology are incorporated into the standard FMEA process (see Fig. 1). The purpose of the planning and preparation step of FMEA is to define what systems, subsystems and components will be studied. In the risk determination step, the causes, modes and effects of the failure as well as how they interact with each other are identified. In the standard FMEA process, the risks are also evaluated during this step. The proposed method is relevant when insufficient prior knowledge is available for direct risk evaluation, or the FMEA experts seek to increase the consistency of their work based on the pairwise comparison of different failure modes. As the proposed method favours comparisons with already evaluated risks, it is worth examining what kinds of risks have been evaluated before the FMEA assessment. Reference risks can be taken from previous FMEA studies or can reflect failures that can be clearly defined and evaluated and identical to the studied problem. Contrary, there will be risks that should be evaluated by the team members. The key idea of the proposed method is that the evaluation of these risks can be highly supported by comparisons (especially with already evaluated risks).

During the scale definition step, the scales used for comparison and evaluation of the risks are specified. During the pairwise comparison step, the estimated risks are compared in terms of severity as well as probabilities of occurrence and detection. The indices of the compared risks that will be transformed into the S, O and D indices of the FMEA scale are provided by the calculation of the importance weights step and is followed by the consistency evaluation step that checks the results of the pairwise comparison. According to the standard FMEA process, multiplication of the identified indices specifies the RPN of the studied failure modes. In the following sections, the details of these steps will be presented with the help of an example to demonstrate evaluation of the O index from a set of known and unknown failures.

### Risk determination

**Fig. 2:**
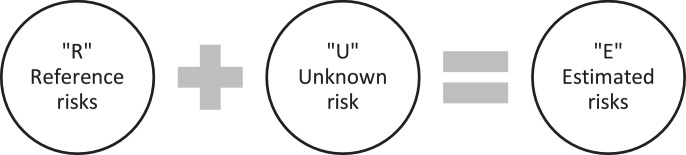
The pairwise comparisons are elaborated on the elements of the unified sets of the reference and unknown risks. The estimated risks which are the inputs of the pairwise comparison included the reference and the unknown risks.

The risk determination step defines the set of unknown risks that should be evaluated ($n_{u}=\left|U\right|$) and searches for similar reference risks in previously finalized FMEA studies ($n_{R}=\left|R\right|$) that can support the mapping of the evaluation (see Fig. 2.). It should be noted that when a new product or process is introduced, it is possible that $n_{R}=0$ as a relevant reference cannot be identified. Although the S, O and D indices can be directly assigned to the risks by estimating their value, a much more accurate estimate can be obtained by considering the subset of risks and comparing them in pairs as a pairwise comparison of the unknown risks still supports such judgments and ensures estimations of risk are consistent.

### Scale definition

The pairwise comparison is based on the tailored version of the fundamental scale of the AHP method [15] (see Table 1).

**Table 1:** The scales defined for pairwise comparisons.

*a*_*i, j*_	Severity	Occurrence	Detectability
0	Equally critical	Equally frequent	Equally detectable
1	Slightly more critical	Slightly more frequent	Slightly less detectable
2	Moderately more critical	Moderately more frequent	Moderately less detectable
3	More critical	More frequent	Less detectable
4	Strongly more critical	Strongly more frequent	Strongly less detectable
5	Strongly more (+) critical	Strongly more (+) frequent	Strongly less (-) detectable
6	Very strongly more critical	Very strongly more frequent	Very strongly less detectable
7	Very strongly more (+) critical	Very strongly more (+) frequent	Very strongly less (-) detectable
8	Extremely more critical	Extremely more frequent	Extremely less detectable
9	Extremely more (+) critical	Extremely more (+) frequent	Extremely less (-) detectable

**Fig. 3:**
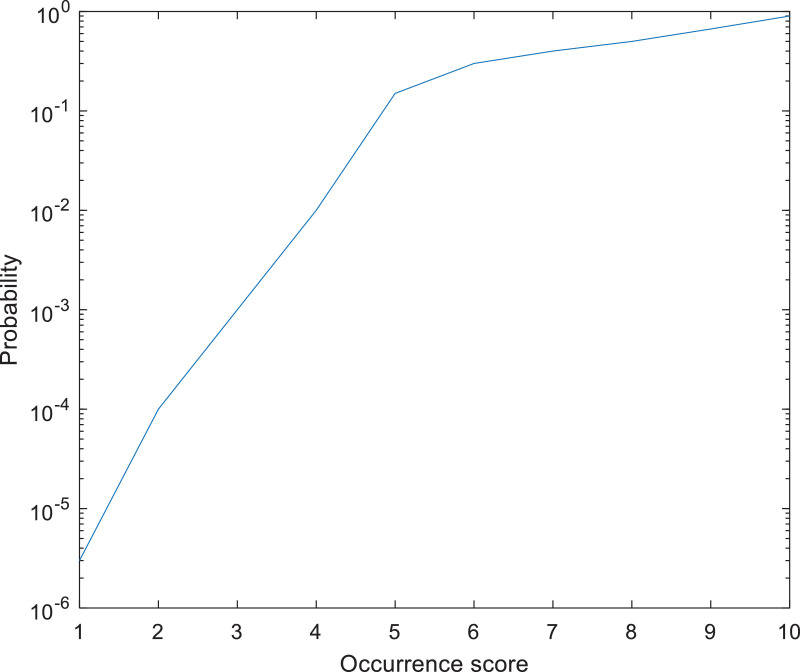
Example of the function of the occurrence scoring according to Table 2 (Adapted from Terninko (2003)).

The comparison can also be supported by visualization of the indexes applied in the FMEA study (see Table 2 and Fig. 3).

**Table 2:** Example of occurrence scoring (Adapted from Terninko (2003)).

Score	Occurrence	Probability
1	Almost Never	3/10^6^
2	Rarely	100/10^6^
3	Very Slight	1000/10^6^
4	Slight	10,000/10^6^
5	Low	150,000/10^6^
6	Medium	300,000/10^6^
7	Moderately High	400,000/10^6^
8	High	500,000/10^6^
9	Very High	666,667/10^6^
10	Almost Certain	900,000/10^6^

This visualization is important as the pairwise comparisons should reflect the differences in the FMEA scorings, as when the *i* th and *j*-th failure modes are compared, $a_{i,j}=x_{i}-x_{j}$ (please note *x_i_* is used to represent the *S_i_, O_i_* or *D_i_* indexes and *a*_*i, j*_ can be negative if the *j*-th index is exceeds than the index of the *i* th failure mode as, $a_{j,i}=-a_{i,j}$.

### Pairwise comparison

Once every $n=\;n_{u}+n_{R}\;$risk has been collected, the FMEA team performs n(n−1)/2 comparisons to obtain the *a*_*i, j*_ *i* *<* *j* values. It is advisable to ensure that the number of evaluations is kept to the minimum to avoid human fatigue. When three or four reference risks and four unknown failure modes are compared, the number of assessments is between 3 and 28, which setup is suggested as an ideal case of the proposed method. The remaining values of the matrix are entered as $a_{j,i}=-a_{i,j}$ which yields the following comparison matrix:

(1) $$A=\left[\begin{array}{cccc} 0 & a_{1,2} & \ldots & a_{1,n}\\ a_{2,1} & \ldots & a_{i,j} & \vdots \\ \vdots & \vdots & \vdots & \vdots \\ a_{n,1} & \ldots & \ldots & 0 \end{array}\right]$$

To demonstrate the proposed method detailed examples are given based on the analysis of the following comparison matrix resulted after the comparisons of the occurrences of six failures:

(2) $$A=\left\lceil \begin{array}{cccccc} 0 & -5 & -1 & -3 & -6 & 1\\ 5 & 0 & 4 & 1 & -1 & 8\\ 1 & -4 & 0 & -3 & -5 & 3\\ 3 & -1 & 3 & 0 & -3 & 6\\ 6 & 1 & 5 & 3 & 0 & 8\\ -1 & -8 & -3 & -6 & -8 & 0 \end{array}\right\rceil$$

### Calculation of the importance weights

The goal is to find the set of priorities $w={\left[w_{1},\ldots ,w_{n}\right]}^{T}$ such that $a_{ij}=w_{i}-w_{j}$ matches the comparisons. The proposed method is easily applicable as the arithmetic mean

(3) $$w_{i}=\frac{1}{n}\sum_{j=1}^{n}a_{i,j}$$

which yields an optimal solution for the additive pairwise comparison problem [16]

(4) $$min_{w}J\left(w\right)=\sum_{i=1}^{n}\sum_{j=1}^{n}{\left(a_{i,j}-\left(w_{i}-w_{j}\right)\right)}^{2}$$

(5) $$s.t.\;\sum_{i=1}^{n}w_{i}=0$$

The application of Eq. (3) in case of the presented example results in: $w_{i}=\left[-2,3333;\;2,8333;\;-1,3333;\;1,3333;3,8333;\;-4,3333\right]$, which reflects that the 6th and 5th failure modes will receive the lowest and highest occurrence index, respectively.

### Consistency evaluation

As in the case of consistency evaluations the transitivity $a_{i,j}=a_{i,k}+a_{k,j}$ should be fulfilled, the average consistency over all triplets when *i* and *j* are fixed is:

(6) $$e_{i,j}=a_{i,j}-\frac{1}{n}\sum_{k=1}^{n}\left(a_{i,k}+a_{k,j}\right)$$

Assuming $a_{k,j}=-a_{j,k}$ and $w_{j}=\frac{1}{n}\sum_{k=1}^{n}a_{j,k}$, the inconsistency can be expressed in the form of the approximation error (see Eq. (7))

(7) $$e_{i,j}=\frac{1}{n}\sum_{k=1}^{n}\left(a_{i,j}+a_{k,i}+a_{j,k}\right)=a_{i,j}-\left(w_{i}-w_{j}\right)$$

which proves that the result $\sum_{i=1}^{n}\sum_{i=1}^{n}{e_{i,j}}^{2}$ of the minimized objective function directly represents the inconsistency of the evaluations.

With the use of the above equation the inconsistencies of the evaluations are the following in the studied example:

(8) $$E=\left\lceil \begin{array}{cccccc} 0 & 0.1667 & 0 & 0.6667 & 0.1667 & -1\\ -0.1667 & 0 & -0.1667 & -0.5 & 0 & 0.8333\\ 0 & 0.1667 & 0 & -0.3333 & 0.1667 & 0\\ -0.6667 & 0.5 & 0.333 & 0 & -0.5 & 0.3333\\ -0.1667 & 0 & -0.1667 & 0.5 & 0 & -0.1667\\ 1 & -0.8333 & 0 & -0.3333 & 0.1667 & 0 \end{array}\right\rceil$$

To obtain a measure that is independent of *n,* the relative error is calculated:

(9) $$RE\left(A\right)=\frac{\sum_{i=1}^{n}\sum_{j=1}^{n}{e_{i,j}}^{2}}{\sum_{i=1}^{n}\sum_{j=1}^{n}{a_{i,j}}^{2}}$$

In general, when RE(A) < 0.1 is acceptable. If the inconsistency is too high, a risk estimation should be calculated again. The *e*_*i, j*_ inconsistency values given can support the reassessment as the nonzero elements of *E* highlight which evaluations are worth to be repeated. The reassessment can be supported by the corrected evaluation matrix by giving hints of corrected evaluations:

(10) Ac=round(A−E)

In case of highly inconsistent result, itis worth considering the formation of a new FMEA team as the results reflect that the members of the FMEA team are not competent to assess the subject of the FMEA.

In the studied example, the sum of squares error is $\frac{1}{n}\sum_{i=1}^{n}\frac{1}{n}\sum_{j=1}^{n}{e_{i,j}}^{2}=0.1667$ and RE(A) = 0.0098 which reflects that the evaluations are consistent, just a fine-tuning of the evaluations are needed

When the suggested corrections calculated by Eq. (10) are accepted, the corrected comparison matrix becomes:

(11) $$Ac=\left\lceil \begin{array}{cccccc} 0 & -5 & -1 & -4 & -6 & 2\\ 5 & 0 & 4 & 1 & -1 & 7\\ 1 & -4 & 0 & -3 & -5 & 3\\ 4 & -1 & 3 & 0 & -3 & 6\\ 6 & 1 & 5 & 3 & 0 & 8\\ -2 & -7 & -3 & -6 & -8 & 0 \end{array}\right\rceil$$

which results even less inconsistency, RE(Ac) = 0.033.

### Determination of the S, O and D indices

As *a*_*i, j*_ reflects the differences between the severity, observability or the detectability of two failure modes, the scores of these variables are also expected to approximate the ratio of the extracted importance weighs:

(12) $$a_{i,j}\approx w_{i}-w_{j}\approx x_{i}-x_{j}$$

When *j* ∈ *R* represents a reference and *i* ∈ *U* denotes an unknown failure mode, then *x_i_* can be estimated as

(13) $$x_{i}=\left(w_{i}-w_{j}\right)+x_{j}$$

In case more references are available the estimation is based on the average of the estimates:

(14) $$x_{i}=\left(w_{i}-\frac{1}{n_{R}}\sum_{j\in R}w_{j}\right)+\sum_{j\in R}x_{j}$$

When a reference is unavailable, instead of $\frac{1}{n_{R}}\sum_{j\in R}w_{j}$, the mean of all the importance weights is used (which is zero) and instead of using ${\bar{x}}_{R}=\sum_{j\in R}x_{j}$, the results are placed into middle of the scale:

(15) $$x_{i}=w_{i}+5$$

For standard FMEA applications the calculated *x_i_* values are rounded to the nearest integer.

**Fig. 4:**
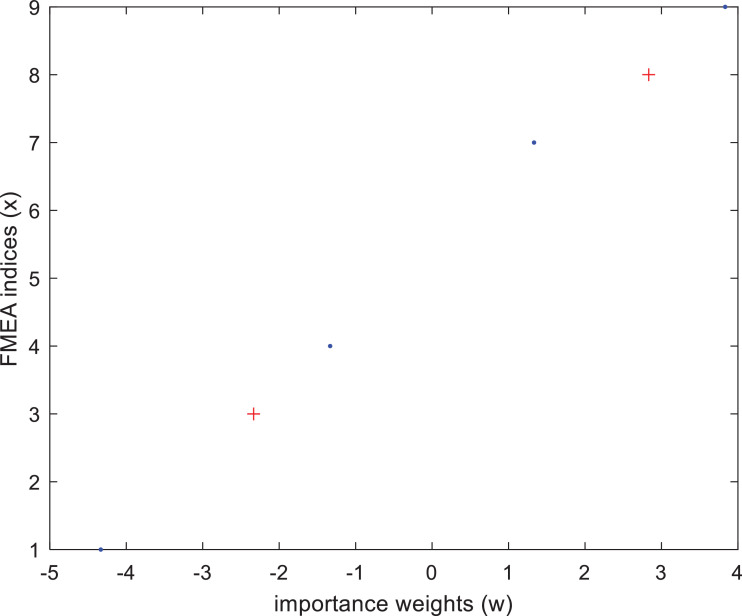
The calculated importance weights and the FMEA indices (red data points represent the referenced failure modes).

In the following, two examples will be given for the determination of the indices. In the first example the occurrences of the first two failures were assumed to be already evaluated (3 and 8) and used as references as depicted in Fig. 4. With the help of Eq. (14) the estimated observation indices of the remaining four unknown risks are [4,^7^,^9^,1] in both cases when the calculations are based on the original *A* and the corrected *Ac* matrices.

In the second estimation example it is assumed that none of the compared failure modes are known. With the help of Eq. (15) the estimated observation indices are [3,^8^,^4^,^6^,^9^,1] which result is almost identical to the case when the references were also applied.

As demonstrated by these examples, the proposed method is intuitive and can be easily applied as well as implemented as only elementary mathematical operations are utilized. The supplementary excel table (available at www.abonyilab.com) and the MATLAB program presented in the appendix demonstrate the applicability of the method. The presented tool also applicable to the evaluation of the inconsistency of existing FMEAs. In this case, the FMEA team reassesses the risks with the proposed pairwise comparison method, checks the consistency of their work and estimates the S, O, D indices. When the pairwise comparison is consistent, and the estimated indices are not identical to the known risks of the FMEA, the existing FMEA is not consistent and appropriate, so it should be revised based on the results of the proposed method.

## Appendix Supplementary materials

Image, application 1

Image, application 2
