# Supplementary material for: Pairwise comparison based failure mode and effects analysis (FMEA)
Source: MethodsX. 2020 Jul 23;7:101007. doi: 10.1016/j.mex.2020.101007 (PMC7397688; doi:10.1016/j.mex.2020.101007)
Supplement: Supplementary file 2 [file mmc2.docx]

**Appendix – Matlab code of the illustrative examples**

%% Initialization

A=[0 -5 -1 -3 -6 1; ...

5 0 4 1 -1 8 ; ...

1 -4 0 -3 -5 3; ...

3 -1 3 0 -3 6; ...

6 1 5 3 0 8 ; ...

-1 -8 -3 -6 -8 0];

n=length(A) %number of comparisions

O=[3 8 4 7 9 1]; %original risks (assumed to be partly known)

ref=[1 2 ]; %indices of the known risks

unknown=setdiff(1:n,ref); %indices of the unknown risks

%% Estimation of the weights and evaluation of the inconsistency

w=mean(A,2)

Ae=(repmat(w,1,n)-repmat(w',n,1))

E=A-Ae

Em=mean(mean((A-Ae).^2))

REA=Em/mean(mean((A).^2))

%% Correction

Ac=round(A-E);

w=mean(Ac,2)

Aec=(repmat(w,1,n)-repmat(w',n,1))

Ec=Ac-Aec

Emc=mean(mean((A-Aec).^2))

REA=Emc/mean(mean((A).^2))

%% Prediction and plot

figure(10)

plot(w(ref),O(ref),'r+');

hold on

plot(w(unknown),O(unknown),'b.');

xlabel('Importance weights (w)')

ylabel('FMEA indices (x)')

if ~isempty(ref)

Oe=(mean(O(ref))+(w-mean(w(ref))))

else

Oe=(5+(w));

end

[O' round(Oe)]
